# Evaluating the Feasibility of a Multiplayer Role-Playing Game as a Behavioral Health Intervention in Adolescent Patients With Chronic Physical or Mental Conditions: Protocol for a Cohort Study

**DOI:** 10.2196/43987

**Published:** 2023-06-27

**Authors:** Dmitriy Babichenko, Ana Radovic, Ravi Patel, Alexis Hester, Koehler Powell, Nicholas Eggers, David Happe

**Affiliations:** 1 School of Computing and Information University of Pittsburgh Pittsburgh, PA United States; 2 School of Medicine University of Pittsburgh Pittsburgh, PA United States; 3 School of Pharmacy University of Pittsburgh Pittsburgh, PA United States; 4 School of Engineering University of Pittsburgh Pittsburgh, PA United States

**Keywords:** role-playing games, social isolation, depression, game-based interventions, development, intervention, game, teen, patient, chronic, mental condition, quality of life, engagement, symptoms, data, clinical

## Abstract

**Background:**

Numerous studies have revealed that adolescents with chronic physical or mental conditions (CPMCs) are at an increased risk for depression and anxiety, with serious direct and indirect negative effects on treatment adherence, family functioning, and health-related quality of life. As game-based approaches are effective interventions in treating anxiety and depression, we propose to explore the use of a multiplayer role-playing game (RPG) as a potential intervention for social isolation, anxiety, and depression.

**Objective:**

The objectives of this study were to (1) determine the feasibility of using Masks, a multiplayer RPG, as an intervention for social isolation, anxiety, and depression in adolescents with CPMCs; (2) evaluate the viability of the research process; and (3) gauge participation in and engagement with RPG-based interventions.

**Methods:**

This study is a remote synchronous game-based intervention for adolescents with CPMCs aged 14-19 years. Eligible participants completed a web-based baseline survey to assess anxiety, depression, and social isolation and to identify their gaming habits. After completing the baseline survey, they participated in 5 moderated Masks game sessions. In Masks, players assume the roles of young superheroes; select their character types, superpowers; and perform actions determined by the game’s rule system and dice rolls. All game sessions were played using Discord, a communication platform commonly used by gaming communities. Games were led and moderated by game masters (GMs). After each game session, participants completed surveys to assess changes in anxiety, depression, and social isolation, and their attitude toward the game and the user experience. The participants also completed an exit survey after all 5 game sessions (modified version of the Patient Health Questionnaire and the Generalized Anxiety Disorder Questionnaire, and 17 open-ended questions). The GMs rated each game session and reported on gameplay, player behavior, comfort, and engagement levels of the players.

**Results:**

As of March 2020, six participants were recruited for the pilot study to participate in moderated web-based game sessions of Masks; 3 completed all game sessions and all required assessments. Although the number of participants was too low to draw generalizable conclusions, self-reported clinical outcomes did seem to indicate a positive change in depression, anxiety, and social isolation symptoms. Qualitative analysis of postgame survey data from participants and GMs indicated high levels of engagement and enjoyment. Furthermore, the participants provided feedback about improved mood and engagement related to weekly participation in Masks. Lastly, responses to the exit survey showed interest in future RPG-related studies.

**Conclusions:**

We established a workflow for gameplay and evaluated a research protocol for evaluating the impact of RPG participation on isolation, anxiety, and depression symptoms in adolescents with CPMCs. Preliminary data collected from the pilot study support the validity of the research protocol and the use of RPG-based interventions in larger clinical studies.

**International Registered Report Identifier (IRRID):**

RR1-10.2196/43987

## Introduction

Numerous studies have revealed that adolescents with chronic physical or mental conditions (CPMCs) are at an increased risk for depression and anxiety, with serious direct and indirect negative effects on treatment adherence, family functioning, and health-related quality of life [1-3]. Regular participation in social activities with peers is particularly crucial in adolescence where it can provide a critical key component for developing healthy psychological development and identity formation [4,5]. Moreover, support from peers can also play a significant role in helping young people with CPMCs cope with illness and with its “inherent psychosocial and lifestyle changes” [6].

A number of studies have shown advantages of web-based socialization and web-based peer support for patients with disorders such as autism [7,8], social anxiety disorder [9], and chronic conditions such as cystic fibrosis (CF), where the danger of cross-infection of respiratory tract bacteria [10-12] prevents patients with CF from socializing in person.

With the outbreak of the COVID-19 pandemic, social isolation has become an even more critical problem, preventing adolescents with CPMCs from socializing not only with their peers but also with family members and other members of their support networks [13-15]. A number of mobile apps and web-based interventions have emerged to help patients with CPMCs to deal with stress [16], social isolation [17,18], anxiety [19], depression [19,20], symptom burden [16], and disease management [21,22]. Although numerous studies have shown a positive impact of these apps [16-18,20-22], none of the app-based interventions that we reviewed had social elements that would allow patients to interact with their peers or members of their support networks. Moreover, we were unable to find the majority of the apps described in the reviewed studies on publicly available app stores such as Apple App Store, Google Play Store, or Steam, suggesting that the apps used as part of research studies either were not available to the general public or did not have long-term support from the developers. Another shortcoming of many app-based interventions is that they target a specific mental or physical health condition (eg, autism [23] and diabetes [21,22]) but do not generalize to broader populations of adolescents with CPMCs.

Several attempts have been made to develop or study game-based interventions for social isolation. The use of video games as mental health interventions has been well explored in recent years [24-26], but, surprisingly, little work has been done to research the use and impact of video games on social isolation. Pearce et al [27], Zhu [28], and Martinez et al [29] explored how individual players as well as entire families leveraged the *Animal Crossing: New Horizons* game to battle pandemic-related social isolation. Udapola [30,31] proposes the use of social virtual reality (VR) platforms as an intervention for socially isolated adolescents with significant illness. Although many studies have found VR interventions to be effective tools for cognitive behavioral therapy [32-34], the availability and cost of VR devices may prove to be a significant barrier to scaling VR-based interventions to large populations of socially isolated adolescents.

However, to the best of our knowledge, there are no studies that explore the use of role-playing games (RPGs) in facilitating adolescents with CPMCs to escape their real world by creating an in-game character that may allow for identity exploration without the limitations of their CPMCs. Furthermore, as of the time of this writing, we were unable to find studies that evaluate the impact of RPG participation on self-reported isolation, anxiety, and depression symptoms in adolescents with CPMCs.

According to the Adventure Game Industry Market Research Summary [35], an estimated 5.5 million people in the United States regularly play RPGs. There is more to RPGs than the sheer number of players—there is strong evidence to suggest that RPGs facilitate the creation and sustainability of game enthusiasts’ communities [36,37]. 

We hypothesize that participation in RPG communities of their peers (other adolescents with CPMCs) may decrease social isolation, increase feelings of acceptance, and enhance peer support for adolescents with CPMCs. The gameplay itself and the community around the game may provide peer support around some of the struggles and difficulties that adolescents with CPMCs have, including physical and mental health maintenance, adherence to complex medication regimens, self-advocacy, reduction in depression and anxiety symptoms, and transition from pediatric to adult health care.

Before studying whether participation in RPG communities provides this benefit in adolescents with CPMCs, it is important to understand whether it is feasible to engage adolescents with CPMCs through RPG communities.

The goals of this pilot work are to (1) understand the feasibility of recruiting adolescents with CPMCs to participate in an RPG game; (2) assess the feasibility of using Masks, a multiplayer tabletop RPG game, to engage adolescents with CPMCs; and (3) develop a research protocol for using and evaluating RPG games as potential clinical interventions for anxiety and depression associated with social isolation.

## Methods

### Initial Recruitment Challenges

We conducted a series of semistructured interviews with 12 patients with pediatric CF (aged 13-18 years) to understand their gaming habits and preferences and gauge their interest in a research study involving RPGs. For this first iteration, we considered setting up a private World of Warcraft (WoW) [38,39] server to allow us to create custom in-game quests for the participating patients with CF. However, 7 of the interviewed patients reported that they did not like WoW or did not want to spend multihour sessions going on quests with strangers. Three of the patients stated that they already spend between 1 and 3 hours per day playing video games and that their parents would object to their participation in a study that requires hours of computer-based gameplay. Lastly, 3 patients indicated that their parents perceived WoW as a violent RPG and would not allow them to participate in such a study.

In a second iteration, we conducted a round of semistructured interviews with a different group of 5 patients with pediatric CF (aged 14-17 years, no overlap with the first group). We asked the participants to describe elements of an RPG that they would be interested in playing. A word frequency analysis of the collected data showed the following words being present in every response: adventure, action, puzzle, superhero. After discussing interview results with several University of Pittsburgh game design instructors, a lead game designer from a Pittsburgh-based game design studio, and with members of 2 student-based RPG clubs, we selected the Masks RPG as a potential intervention for this study.

Our original Masks RPG-based study design called for weekly 3-hour game sessions. Once we began the recruitment phase, we focused on the population with pediatric CF. A total of 11 patients with CF expressed interest in participating in the study. However, during the informed consent process, once they understood the required time investment, 7 participants withdrew before being consented. Either the participants or their parents expressed concerns over the required time commitment. The remaining 4 participants were not able to find a common weekly 3-hour slot that would work for the entire group.

In our next recruitment iteration, we expanded the participant inclusion criteria to any pediatric onset chronic disease that requires rigid medication or treatment adherence, including CF, type 1 diabetes, sickle cell anemia, and pediatric cancers that require maintenance chemotherapy. From this population, we recruited and consented 8 participants and scheduled weekly Masks game sessions. A total of 6 of the 8 participants left the study after the first 2 game sessions, stating scheduling difficulties and interference with school activities as primary reasons for leaving the study.

On the basis of the recruitment and retention difficulties described above and the feedback received from participants who left the study, we redesigned the study workflow, shortened gameplay to approximately 1.5 hours per game session, and expanded participant inclusion criteria to all adolescents with CPMCs aged 14-19 years. It is important to note that shortening the gameplay sessions did not alter the game scenarios—we split scenarios between multiple game sessions, with each subsequent session’s scenario, activities, and storylines picking up where the previous session left off.

### Study Design

**Figure 1:**
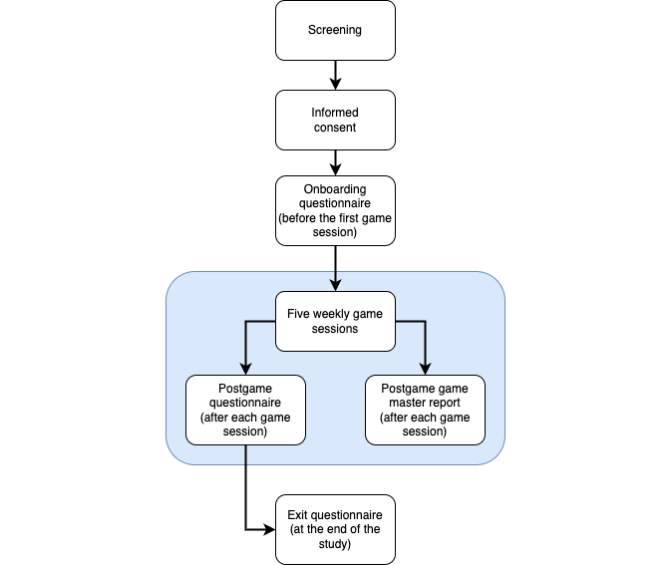
Study design flow diagram.

After the initial screening, participants completed an onboarding questionnaire to collect basic demographic information, as well as baseline data related to depression, social isolation, social support, and symptom burden. Questions included a modified version of the Patient Health Questionnaire omitting the last suicidality question due to anonymity and difficulty reaching participants who express risk (PHQ-8) [40], the Generalized Anxiety Disorder Questionnaire (GAD-7) [41], Social/Informational Support section of the RAND Social Support Survey Instrument (SSSI) [42], and the modified 4-question version of the revised UCLA Loneliness Scale (LS) [43-45]. PHQ-8, GAD-7, SSSI, and LS are extensively validated instruments that have shown good reliability, validity, and adaptability for patients across multiple research studies (Figure 1).

After completing the onboarding questionnaires, participants played a series of 5 weekly games, with the average game session duration of 1.5 to 2 hours.

Each game session was guided by 2 game masters (GMs). The role of a GM is to guide the narrative, environments, and the non-player aspects of the game, as well as provide guidance to players and address any player disputes. In the context of this study, the GMs also played an additional role of field researchers, recording their perceptions of how the players interacted with each other and with the game, whether players were engaged, and whether players discussed their CPMC symptoms or their personal lives with each other.

All GMs who helped facilitate this study were University of Pittsburgh students. At the time of the study, 2 of the GMs were second-year students at the School of Medicine, one was a second-year student at the School of Pharmacy and one a third-year student at the School of Engineering. All 4 GMs were selected based on 2 criteria: prior GM experience with tabletop RPGs such as Dungeons & Dragons (D&D) and prior academic research experience in a university setting. As part of the screening, all GMs had to learn how to plan and execute a Masks game session with this project’s investigators as players. Once the GMs demonstrated their ability to plan, organize, and run game sessions, they were required to complete the Responsible Conduct of Research, Conflict of Interest, and Human Subjects Research (both biomedical and social/behavioral/educational) web-based training modules required by the University of Pittsburgh Human Research Protection Office.

Throughout the study, the GMs were responsible for planning, scheduling, and running game sessions, as well as for ensuring that participants complete postgame surveys.

The GMs rated each game session on the scale of 1 to 10 (1=worst and 10=best), provided their opinion on what could have made the session better, described any issues with gameplay or with player behavior, and provided comments on comfort and engagement levels of the players.

At the end of each game, participants completed a 27-question Likert questionnaire (Textbox 1) to collect feedback on the game session. Questions focused on participants’ interactions with other players and with the GM, as well as their opinions of the storyline, how the game was handled, and whether the game was engaging or entertaining.

Lastly, the participants completed an exit questionnaire after they concluded all 5 game sessions. The exit questionnaire contained a modified version of the PHQ-9 and GAD-7 questionnaires. Additionally, the exit questionnaire also contained 17 open-ended questions (Textbox 2), such as “What was the most frustrating moment or aspect of the game experience?” and “If you had a magic wand to wave, and you could change, add, or remove anything from the game experience, what would it be?”

**Postgame questionnaire (responses for the questions are on the scale of 1 to 5: 1=strongly disagree, 2=somewhat disagree, 3=neither agree nor disagree, 4=somewhat agree, 5=strongly agree).**

1. I enjoyed playing the game tonight
2. I would like to play the game again
3. I felt emotionally safe playing the game
4. I felt the other players treated me with respect
5. I felt the other players treated each other with respect
6. I felt the other players were kind to me
7. I felt the other players were kind to each other
8. I felt comfortable talking to the other players
9. I was interested in what the other players had to say
10. I would like to get to know the other players more
11. Playing this game made me feel happy
12. Playing this game made me feel less anxious
13. Playing this game made me feel less depressed
14. Playing this game made me feel less alone
15. I felt comfortable talking about my experience with chronic physical or mental conditions (CPMCs)
16. I liked talking about my experience with CPMCs
17. I liked that I was able to play with other people with CPMCs
18. I liked that I was able to talk with other people with CPMCs
19. I am looking forward to the next gaming session
20. The game master was helpful
21. The game master made me feel safe
22. The game master talked too much
23. The game master talked just right
24. The game master talked too little
25. I liked having the game master present
26. I like the game Masks
27. I like role-playing games

**Masks research study exit questionnaire, open-ended questions.**

1. What was the most frustrating moment or aspect of the game experience?
2. What was your favorite moment or aspect of the game experience?
3. Was there anything you wanted to do that you couldn't?
4. If you had a magic wand to wave, and you could change, add, or remove anything from the experience, what would it be?
5. What were you doing in the experience?
6. What was your role?
7. Why did you choose this particular role?
8. How would you describe this game to your friends and family?
9. Did you talk to your parents about playing this game?
10. Did your participation in this game interfere with other activities?
11. Did your parents mind that game sessions took 3 hours to complete?
12. Why did you agree to participate in this study?
13. Was it because of the pay, or were there any other reasons?
14. Did you ever feel uncomfortable during any of the game sessions? If so, why?
15. During the games, did you discuss your symptoms with any other players?
  - What were these discussions like?
  - What specifically did you discuss?
16. How did participating in these games make you feel?
17. Would you be interested in continuing playing this game or other games like it in the future, outside of this study?

### Study Population

For the purposes of this study, we defined CPMC as any physical or mental condition where the symptoms have persisted for at least 1 year and where the participant has been receiving treatments from health care professionals for a period of at least 1 year. We required that each participant have consistent access to a computer and could demonstrate native-like fluency in the English language. Participants with impairments that prevent them from using a computer were excluded from the study because it would not have been possible for them to interface with the study materials.

### Ethics Approval

#### Ethics Review

This study was reviewed and approved by the University of Pittsburgh Human Research Protection Office (protocol number STUDY19090313, “Multiplayer RPG as depression/isolation intervention”).

#### Informed Consent

After the initial screening phone call with the participant and parent dyad, the research team emailed the consent form to the parent and the participant for review and to have as a reference at the time of the consent form review. A research team member met with both the parent and the participant via a Zoom conference call to review the consent form and to answer any questions. The consent form included general explanations of what research is, specific information describing the study, explanation of inclusion criteria and how they relate to the specific participant being consented, detailed explanation of the game, participation requirements, potential benefits, and possible risks and discomforts. After the consent form review and after the parents and the participants confirmed that they understand the information and do not have additional questions, we asked the parents and the participants to digitally sign the consent form via the DocuSign electronic signature service. The final signed copy was automatically emailed to all parties and stored on the University of Pittsburgh DocuSign service cloud.

#### Privacy Protection

This study did not collect any identifiable data from the parents or the participants except for the data required for the consent forms. Signed consent forms were automatically stored on the University of Pittsburgh DocuSign service cloud, accessible only to the study’s principal investigator. After the consent process, participants were identified only by their chosen game character names, with no links to their names or their Health Insurance Portability and Accountability Act–protected information. Transcripts from game sessions (ie, Discord chat transcripts) and all raw survey data were reviewed after each game session by at least 3 investigators to ensure that no identifiable data were entered. Coded data from interviews, game transcripts, and survey responses were stored in a secure University of Pittsburgh–approved Microsoft OneDrive folder accessible only by the investigators.

#### Compensation

Participants were compensated US $20.00 per each completed game session and an additional US $20.00 for completing all the questionnaires associated with the study.

### Intervention

Masks [46] is a tabletop role-playing system produced by Magpie Games. In Masks, players assume the roles of young superheroes in a team (such as the Teen Titans or the X-Men) as they come of age in the face of superheroic trials and tribulations.

A tabletop RPG is an RPG in which the players “describe their characters' actions through speech” and determine the actions of their characters based on their character sheets. The outcomes of the characters’ actions are determined by a formal system of rules and guidelines. “Within the rules, players have the freedom to improvise; their choices shape the direction and outcome of the game” [47].

The players select playbooks [48], which are based around the type of superhero they want to be and create their own characters to play as. They are similar to other tabletop RPGs like D&D, because the key element of gameplay is acting as a character in scenarios designed and led by a GM. By acting as their characters in response to these scenarios, the group creates a narrative that they experience together. Dice and other tools to decide outcomes are used to add an element of unpredictability to the narrative and aid in the improvisation process.

Unlike most other RPGs, Masks’ mechanics are more focused around a character's emotional state and personal development. For example, the Label system explains mechanically how the characters grow into their powers based on the realizations they make about themselves and the influence of others around them. Instead of taking damage in ways that could lead to their characters dying, the system focuses more on the feelings that challenges and enemies can impose on the characters. A character who fails to use his or her powers against a formidable opponent in a stressful situation could feel insecure or, potentially, angry. By focusing on emotional states rather than physical health, players are free to invest in their characters without fear of them dying, while being given opportunities to create dramatic and narrative tension.

To ensure that players are kept comfortable enough to take creative risks and better tell the story they want to tell, it is very important that practices such as the “Blackcard” be used to ensure that every player is having fun. The “Blackcard” is a sign that can be raised if anybody in the party is feeling uncomfortable out of character about something in the game. If it is raised, the game will stop, and the situation will be resolved out of character before play resumes.

### Game Sessions

At the beginning of the study, we conducted game session 0 (zero), where the GMs explained the game mechanics to the players and the players selected their in-game characters. We used the *MASKS: A New Generation* core book (purchased directly from Magpie Games) for the world, scenario, rule descriptions, and character sheets [48]. Players selected their in-game characters from the list of predefined characters described in the core book (Table 1).

**Table 1:** Character types selected by the participants.^a^

Participant (in-game alias)	Character type	Abilities
Kichi Ito	The Delinquent	(1) Teleportation; (2) tricks illusions; (3) gadgetry and hacking; and (4) psychic weapons, emotion control, and power negation
Rio	The Bull	Someone or something changed you, made you into a perfect weapon: superhumanly tough, incredibly strong, and uniquely skilled at ﬁghting. Decide how each of those abilities manifests
Ryder	The Janus	(1) Rodent or insect control; (2) one generation, venom, or webs; (3) energy absorption; (4) supernatural senses; (5) impossible mobility; and (6) substance mimicry

^a^PHQ-9: Patient Health Questionnaire.

Every subsequent game session began with a recap of the events from the previous session. According to participants’ and GMs’ feedback, this was helpful for the group as it kept everyone on the same page and ensured that no one had forgotten anything major from the previous sessions' storylines or activities.

After the recap, the GM described the setting for the current scene, and the players responded by describing their characters’ actions and reactions, causing the scene to evolve and to change. If a character performed an action where the outcome was uncertain, the player had to roll the dice to find out what the character's action (or reaction) would be.

At the end of every session, the GMs debriefed the players by asking them to describe how the events of the session changed each character’s opinion of themselves or of the team they are a part of. Afterward, the GMs asked for comments, questions, and concerns and asked the players if there was anything that they wanted to see more of or less of in the game.

### Remote Play Tools

Despite “tabletop” being in the name, physical proximity is not required to play Masks. Games can easily be conducted using chat with tools such as Discord [49], allowing for play anywhere with reliable internet access.

Discord is a free voice, video, and text chat application with both mobile and desktop clients. Discord allows its users to create private, invite-only virtual servers, a feature that helps ensure participants’ privacy and provides researchers with full control over the conversations and the interactions. We chose Discord over comparable communication tools such as Slack or Microsoft Teams because of its popularity in web-based gaming communities [50], availability of clients for all major operating systems (ie, MacOS, Microsoft Windows, iOS, and Android), and its documented use in multiple research studies [51-54].

For this study, we created a private server accessible only to the research team members and the participants (players). We used the text chat function in this server to coordinate scheduling and the voice chat functionality to conduct game. Although Discord can also display video during voice calls, we disabled that functionality to protect participants’ privacy.

Because Masks requires each player to roll a pair of 6-sided dice, we used M.A.D.D.I.E. (“Masks Automated Discord Dice Interpreter & Explainer!”), a user-created Discord chatbot to simulate dice rolls and facilitate game mechanics. In a dedicated text channel accessible within Discord, M.A.D.D.I.E responds to a concise syntax with both the requested dice rolls and relevant portions of the playbook describing its consequence, reducing the need for manual referencing, and allowing for smoother gameplay.

Lastly, in lieu of having each participant maintain a physical character playbook, we used a Google Drive–hosted common player sheet for character creation and tracking [55].

### Data Analysis Methods

It is important to note that this pilot feasibility study had only 3 participants and that the statistical methods described in this section were initially chosen with a larger study in mind.

To determine how the participants’ perceptions of the game and self-reported anxiety and depression scores changed over time, we calculated the change in per-question response score from session to session, as well as the overall mean response change per participant. When more data are collected from future studies, we will use the Wilcoxon signed rank test to compare both before and after changes in assessment measures.

Because of the small number of participants who participated in this study, we decided to report the participants’ and the GMs’ paraphrased or directly quoted responses. In future iterations of this study, we plan for analyzing participants’ open-ended text responses using inductive theme analysis.

## Results

### Overview

As of March 2020, we recruited 6 adolescents with CPMCs aged 14-19 years through the UPMC Children’s Hospital of Pittsburgh and the ETUDES Center (the Center for Enhancing Triage and Utilization for Depression and Emergent Suicidality in Pediatric Primary Care) [56].

Of the 6 consented participants, 4 participated in the initial game sessions and 3 completed all game sessions and all required surveys.

The 3 participants who participated in all game sessions had the mean and median age of 17 years. Participants identified their gender as cisgender male, transgender female, and nonbinary assigned female at birth. Two of the participants were White, and one was African American. Two of the participants completed 11 grades of high school, and one participant recently graduated from high school (completed 12 grades).

The participants’ self-reported CPMCs included sensory processing disorder and diabetes, and all 3 participants indicated that they need or use more medical care, mental health, or educational services than is usual for most children their age. All participants reported that they have at least 5 people in their immediate support network who provide emotional support “most of the time” to “all of the time.” PHQ-8, GAD-7, SSSI, and LS scores at the time of onboarding are reported in Table 2.

All participants indicated that they prefer to play with other people rather than alone, on average playing between 10 and 20 hours per week, with game genre preferences leaning toward action, adventure, and strategy or puzzles. All participants had prior experience with video game or tabletop RPGs, including games such as D&D, Minecraft [57], Stardew Valley [58], and Undertale [59].

Even though we did not have enough participants in this pilot study to measure the impact of participation in Masks on the participants’ quality of life and mental health with any statistical significance, it is worth mentioning that PHQ-9 and GAD-7 scores we did collect indicated a reduction of depression and anxiety symptoms. Moreover, the postgame questionnaire results showed a consistent positive change in the participants’ opinion of the game from one game session to the next.

**Figure 2:**
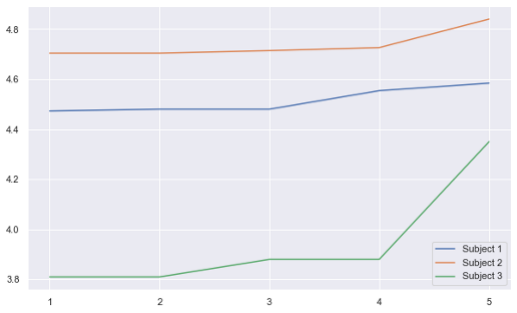
Overall mean change in participants’ engagement and comfort with the game over the course of 5 sessions.

Responses to the postgame questionnaire (Textbox 1) indicated a positive trend in participants’ opinions of and engagement with Masks, individual game sessions, and their comfort levels with other players and with the GMs. Figure 2 shows the trend in the mean response change to the postgame questionnaire over the course of 5 sessions.

In the exit questionnaire, all 3 participants reported that they talked to their parents and friends about the game and the study, that their participation in game sessions did not interfere with other activities, and that their parents did not mind that each game session took several hours to complete.

One participant reported that initially they had difficulties following the story. Another participant reported that at first, they “had a really hard time talking [...] and wanted to stay quiet.”

Favorite aspects of the game included creative environments, unpredictable situations, and doing “something stupid as a team and [working it out together].”

All 3 participants indicated that there was never a situation in the game where they wanted to do something but could not either because of the game environment constraints or due to any issues with other players or the GMs.

To the question of “Why did you choose your particular role?” one participant responded, “I've always enjoyed playing the 'bad boy' type characters,” while another indicated that “[the role] is similar to how I see myself.”

All 3 participants indicated that the game was fun to play and that the characters and other players were “comfortable” and “relatable,” and only 1 participant reported being initially too nervous to talk. None of the participants discussed their physical or mental health during game sessions.

All 3 participants reported that they initially agreed to join the study because of the compensation but “ended up having a really good time.”

It is also worth noting that to the question “Would you be interested in continuing playing this game or other games like it in the future, outside of this study?” all 3 participants answered “Yes.”

Lastly, all participants indicated that playing the game made them “happy” and “excited.” To quote one of the participants, “It made me very happy. I felt safe and it was a lot of fun. It was one of my favorite parts of the week. I looked forward to it every week.”

**Table 2:** Participants’ PHQ-9^a^, GAD-7^b^, LS^c^, and SSSI^d^ scores from the onboarding questionnaire.

Participant (in-game alias)	PHQ-8 score	GAD-7 score	LS score	SSSI score
Kichi Ito	10	2	40	9
Rio	18	5	30	10
Ryder	11	3	23	8

^a^PHQ-9: Patient Health Questionnaire.

^b^GAD-7: Generalized Anxiety Disorder Questionnaire.

^c^LS: UCLA Loneliness Scale.

^d^SSSI: RAND Social Support Survey Instrument.

### GM Postgame Reports

The GMs reported minor technical problems, the most common being connection issues to Discord servers. The GMs did not report any behavior issues (such as toxic behavior or negative language) among the players. The only reported minor issues included lateness to a game session from one player. After the first session, the GMs reported that one player did not feel comfortable and was shy throughout the gameplay. The players did not discuss their personal lives or any topics related to their physical or mental health in any of the game sessions.

## Discussion

In this paper, we presented our pilot work on (1) determining the feasibility of using Masks, a multiplayer RPG, as an intervention for social isolation, anxiety, and depression in adolescents with chronic physical or mental health conditions; (2) evaluating the viability of the research process; and (3) gauging participation in and engagement with RPG-based interventions.

We found that recruiting adolescents to participate from clinical settings was difficult despite study design changes. These recruitment difficulties were mainly due to scheduling difficulties and dedicated time requirements.

For those participants who did participate, we were able to establish a workflow for gameplay and successfully conducted 5 sessions. Participants overall reported satisfaction and enjoyment with gameplay but did not engage in discussing their CPMCs during play as hypothesized. Of particular interest were participants’ preferences to play with other people rather than alone and participants’ prior experience with multiplayer games. These findings correspond to findings in other studies related to game-based interventions [60-63], suggesting that participants with prior multiplayer game experience are more likely to participate in game-related studies. These findings also suggest that in future iterations of this study we will attempt to recruit adolescents with CPMCs from multiplayer game communities rather than from clinical settings.

Although the number of participants was too low to draw generalizable conclusions, self-reported clinical outcomes did seem to indicate a positive change in depression, anxiety, and social isolation symptoms. Furthermore, the participants provided feedback about improved mood and engagement related to weekly participation in Masks.

It is also important to note that all participants reported that they talked to their parents and friends about the game and the study. Several studies suggest that parents or caregivers of children with CPMCs perceive children’s distress symptoms (anxiety, depression, and physical symptom burden) differently, with parents or caregivers consistently rating the children’s symptoms lower than the children [64-68]. In future iterations of this study, we will explore gameplay design features that could facilitate and promote communications between children and parents or caregivers.

In discussing this work, it is important to acknowledge its limitations. One of the main limitations of this study was recruitment and retention. In the initial phases, we attempted to recruit from clinical practices and hospital settings, but because of scheduling difficulties, dedicated time requirements, and school and family obligations, most participants dropped out of the study. Because of time requirements and scheduling constraints, participants participated in only 5 consecutive game sessions of the planned 10. Another limitation was that one participant did not complete all the questionnaires despite multiple reminders from the GMs and the research team. It is also worth noting that we do not know how the fact that the GMs and the players are all strangers to one another affects interaction and gameplay dynamics compared with a typical game of Masks where the players have had prior social interaction. Although our limited data show that the participants reported being comfortable with the GMs and each other, it would be important to empirically compare a game dynamics and comfort levels of players who are strangers to each other versus players who know each other outside of the gameplay world. Lastly, we planned to compare pre- and post-LS and SSSI scores to determine if the scores have improved over time with each game session. However, we were unable to compare these scales with poststudy values because of accidental omission of including them in the exit questionnaire.

Although the small number of participants (n=3) does not allow us to make generalizable conclusions about the efficacy of RPG-based interventions, we did establish a workflow for gameplay and evaluated a research protocol for evaluating the impact of RPG participation on isolation, anxiety, and depression symptoms in adolescents with CPMCs. It is also worth noting that many recent and past research studies focused on developing custom interventions for a specific health or mental condition. From our own experience [69-72], we know how expensive and time-consuming it is to develop game-based interventions and how difficult and costly they are to maintain and support. This work strongly suggests that it is possible to take a widely available and relatively inexpensive multiplayer tabletop RPG, one that already has an established support network and gamer community, and use it as a possible intervention for social isolation.

Many support groups, community centers, and even entire neighborhoods already have established community game events (eg, game nights and game jams), with a positive impact on player engagement, mental health, and community building [73-76]. If future studies with larger patient samples show significant short- and long-term improvement in social isolation, depression, and anxiety symptoms, clinical practices that provide health care services to adolescents with CPMCs should consider either partnering with existing gamer communities or establishing their own consistently recurring multiplayer RPG events for their patients. Such “game nights” could potentially be used as a supplement to existing group therapies.

Lastly, we believe that the preliminary data collected from the pilot study support the validity of the research protocol and the use of RPG-based interventions in larger clinical studies.

## Abbreviations

CF: cystic fibrosis
CPMC: chronic physical or mental condition
D&D: Dungeons & Dragons
ETUDES: Center for Enhancing Triage and Utilization for Depression and Emergent Suicidality in Pediatric Primary Care
GAD-7: Generalized Anxiety Disorder Questionnaire.
GM: game master
LS: UCLA Loneliness Scale.
M.A.D.D.I.E.: Masks Automated Discord Dice Interpreter & Explainer!
PHQ-8: eight-item Patient Health Questionnaire depression scale
RPG: role-playing game
SSSI: RAND Social Support Survey Instrument.
VR: virtual reality
WoW: World of Warcraft

## Data Availability

The data sets generated or analyzed during this study are available from the corresponding author on reasonable request.
